# Opportunities for More Aging and Disparities Research, Mentoring, and Data Sharing With REGARDS

**DOI:** 10.1093/geroni/igaa057.3155

**Published:** 2020-12-16

**Authors:** Suzanne Judd, Virginia Howard, Mary Cushman, Jennifer Manly, George Howard

**Affiliations:** 1 University of Alabama at Birmingham, Birmingham, Alabama, United States; 2 University of Vermont, Colchester, Vermont, United States; 3 Columbia University, New York, New York, United States

## Abstract

One of the benefits of this large, national cohort study with black and white participants is the opportunity to involve a breadth of researchers in the science. REGARDS actively solicits and engages early career and minority investigators to lead or participate in manuscripts as well as ancillary studies. REGARDS has provided opportunities for >175 ancillary studies, including those that enrich existing outcomes, provide new outcomes, assess new exposures, link with other national data, support extended analyses, and assess genetic associations. Over 70% of the 500+ publications to date have a lead author who was not a funded REGARDS investigator. In this presentation, we will discuss some of the innovative ancillary studies and high-impact manuscripts that have grown out of REGARDS, the processes for developing an ancillary study/manuscript, and the procedures for obtaining REGARDS data. We will describe opportunities for mentored research for junior investigators, as well as independent research projects.

